# Characterization of Bacterial Differences Induced by Cleft-Palate-Related Spatial Heterogeneity

**DOI:** 10.3390/pathogens11070771

**Published:** 2022-07-05

**Authors:** Fangjie Zhou, Zhifei Su, Qinyang Li, Renke Wang, Ying Liao, Min Zhang, Jiyao Li

**Affiliations:** 1 State Key Laboratory of Oral Diseases, National Clinical Research Center for Oral Diseases, West China Hospital of Stomatology, Sichuan University, Chengdu 610000, China; 2020224035150@stu.scu.edu.cn (F.Z.); suzhifei@stu.scu.edu.cn (Z.S.); 2021224035046@stu.scu.edu.cn (Q.L.); wangrenke323@163.com (R.W.); 2 Department of Cariology and Endodontics, West China Hospital of Stomatology, Sichuan University, Chengdu 610000, China; 3 Department of Pediatric Dentistry, Nanjing Stomatological Hospital, Medical School of Nanjing University, Nanjing 210000, China; liaoqiaoling@163.com

**Keywords:** cleft palate, oral bacteria, nasal bacteria, 16S ribosomal RNA, bacterial translocation, spatial heterogeneity, high-throughput sequencing

## Abstract

**Background**: Cleft palate (CP) patients have a higher prevalence of oral and respiratory tract bacterial infections than the general population. Nevertheless, characteristics of bacterial differences induced by CP-related anatomical heterogeneity are unknown. **Methods:** In this study, we systematically described the characteristics of bacteria in the oral and nasal niches in healthy children, CP children, healthy adolescents, CP adolescents, and postoperative adolescents by 454-pyrosequencing technology (V3–V6) to determine bacterial differences induced by CP. **Results:** Due to the CP-induced variations in spatial structure, the early establishment of microecology in CP children was different from that in healthy children. Nasal bacterial composition showed greater changes than in the saliva. Moreover, such discrepancy also appeared in CP and postoperative adolescents who had even undergone surgery > 10 years previously. Interestingly, we found by Lefse analysis that part of bacterial biomarkers in the nasal cavity of CP subjects was common oral flora, suggesting bacterial translocation between the oral and nasal niches. Therefore, we defined the oral–nasal translocation bacteria as O-N bac. By comparing multiple groups, we took the intersection sets of O-N bacs selected from CP children, CP adolescents, and postoperative adolescents as TS O-N bacs with time–character, including *Streptococcus*, *Gemella*, *Alloprevotella*, *Neisseria*, *Rothia*, *Actinomyces*, and *Veillonella*. These bacteria were at the core of the nasal bacterial network in CP subjects, and some were related to infectious diseases. **Conclusions:** CP would lead to significant and long-term differences in oral and nasal flora. TS O-N bacs migrating from the oral to the nasal might be the key stone causing nasal flora dysbiosis in the CP patients.

## 1. Introduction

Cleft palate (CP) is a common congenital defect with approximately 0.2% incidence, which might cause bacterial disorders and a higher prevalence of infectious complications in the respiratory tract and oral cavity [1]. Accumulating evidence has demonstrated that the incidence of respiratory-tract-related infectious diseases is high in patients with maxillofacial dysplasia. The incidence of lower respiratory tract infections in infants with cleft lip and palate (11.9%) and cleft lip (14.3%) is higher than that in normal children [2]. CP is also associated with an increased risk of hospitalization for bronchiolitis [3]. Moreover, studies have shown that CP patients suffer higher caries incidence [4,5,6,7,8], poorer periodontal conditions, and more severe gingivitis [9,10]. It has been demonstrated in numerous studies that CP patients might suffer from a higher prevalence of infectious complications which bacteria contribute to [11,12]. The anatomical structure and functions of the oral and nasal cavity are affected by CP [13]. As anatomical rearrangement might greatly affect the characteristics of flora by changing the microenvironment [14], CP-related changes in anatomical niches might significantly influence the oral and nasal bacteria. Culture and sequencing methods have demonstrated an increased proportion of opportunistic pathogens in the oral cavity of CP patients, such as *Staphylococcus aureus* and *Klebsiella pneumoniae*, with a disrupted function of the bacterial community [12,15,16,17,18].

Cleft palate repair can restore the normal structure and function of the oronasopharynx by closing the defect, which will restore the ecological environment and influence the composition of bacteria [19]. However, the incidence rate of otitis media and respiratory tract infection in postoperative population is still high [20,21]. Few studies have explained such a phenomenon from the perspective of bacterial infection. Moreover, few studies have ever systematically explored the characteristics of CP-related changes in oral and nasal flora.

Ectopic colonization of opportunistic pathogens caused by oral flora migration may be an important cause of some medical conditions, such as atherosclerotic disease, rheumatoid arthritis, inflammatory bowel disease, and colorectal cancer [22,23]. In addition, the cleft palate increases the opportunity for oral and nasal microbial communication. In a previous study, Zhang et al. have briefly described the abnormal migration of microorganisms between the two cavities [11]. However, more in-depth and comprehensive research needs to be carried out. 

The present study aimed to systematically describe changes in CP-related oral and nasal bacteria and explore the possible causes of the high incidence of infectious diseases in CP and postoperative individuals from a bacteriological aspect. We hypothesized that CP establishes a pathological channel between the oral and nasal cavities, increasing the possibility of bacterial translocation between these two ecological niches. 454-pyrosequencing technology was used to systematically explore the characteristics of microbial flora changes in CP patients. Meanwhile, we also identified TS O-N bacs, a group of oral–nasal translocation bacteria detected in CP children, CP adolescents, and postoperative adolescents, which might partly explain CP-related infectious diseases.

## 2. Results

### 2.1. Differences in Oral and Nasal Bacterial Communities between Healthy and CP Children

Salivary and nasal samples were collected from 10 CP children and 10 matched healthy children. There were no significant differences in mean age, gender composition, and tooth eruption status between the two groups (Table S1). 

#### 2.1.1. Effect of CP on Nasal Bacteria in Children Was Greater than That of Oral Bacteria

**Figure 1:**
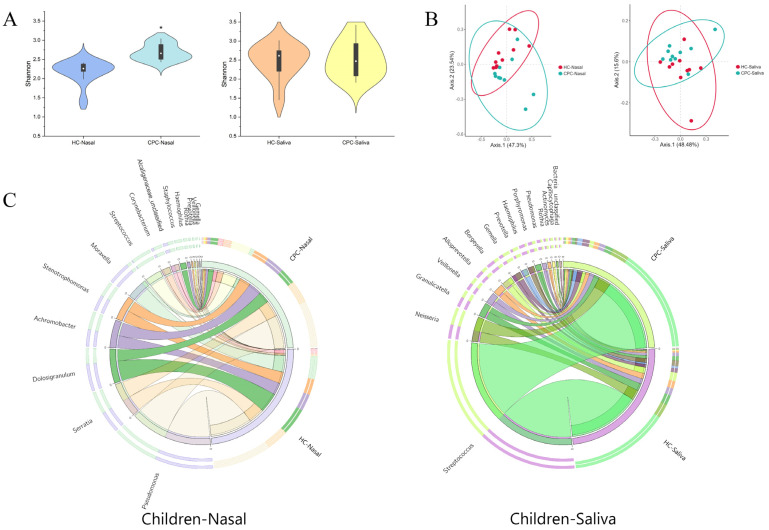
Characteristics of oral and nasal bacterial community in CP children and healthy children. (**A**) Shannon index and (**B**) Principal coordinate analysis based on Bray–Curtis of salivary and nasal bacteria in the CP and healthy children. (**C**) The circos demonstrates the community structure via the relative abundance of the top 15 genus of salivary and nasal bacteria in the two groups. The *p* values were obtained by analysis of variance (* *p* < 0.05). HC: healthy children. CPC: cleft palate children.

Ace, Chao 1, and Shannon indexes showed a significantly higher bacterial abundance and diversity in the nasal samples in CP children (Chao 1 index: 140.20 vs. 114.60, *p* < 0.05; Shannon index: 2.68 vs. 2.18, *p* < 0.05), with no difference in salivary samples between the two groups (Figure 1A and Table S2). The principal coordinates analysis (PCoA) demonstrated a separate trend in nasal bacteria in the two groups, with no apparent trend in the saliva (Figure 1B). These findings suggested that CP-related spatial heterogeneity could induce a greater impact on nasal bacterial diversity than the saliva in children. 

#### 2.1.2. Cleft Palate Changed the Composition of Oral and Nasal Flora in Children 

**Figure 2:**
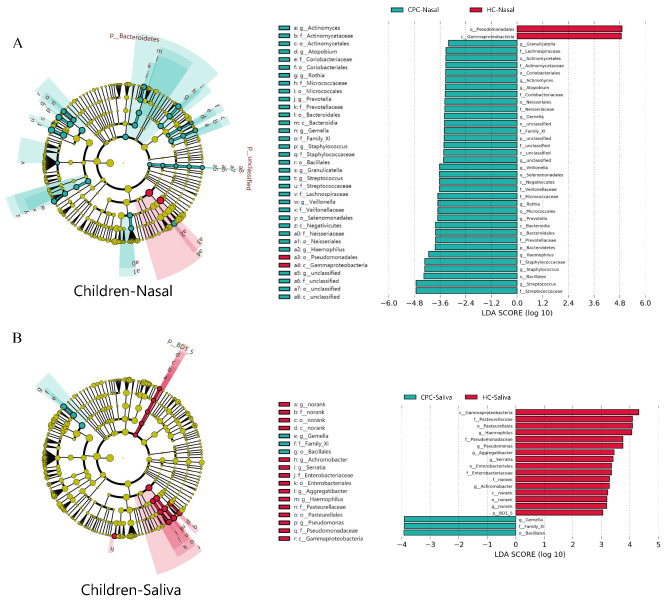
Distinct genera between the CP and healthy children. (**A**,**B**) Linear discriminant analysis effect size (LEfSe, α = 0.05, log_10_ LDA score > 2.0) revealed 10 genera (green) with greater relative abundance in the cleft palate nasal bacteria and 5 genera (red) with greater relative abundance in the control children saliva. H: healthy. CP: cleft palate. Post: postoperative.

The distribution of the high relative abundance of bacteria at the genus level in the nasal cavity of the two groups is described in Figure 1C. The top three genera in the nasal cavity in relative abundance were *Pseudomonas*, *Dolosigranulum*, and *Moraxella* in healthy children, with *Pseudomonas*, *Serratia*, and *Streptococcus* in CP children. The communication between the oral and nasal cavities caused by CP provided more possibilities for the translocation of bacteria between these two ecological niches. To further explore CP-related bacterial changes, linear discriminant analysis effect size (LEfSe) was performed based on the linear discriminant analysis (LDA) score. The results identified special bacterial biomarkers at the genus level of CP/healthy children (LDA score > 2.0, *p* < 0.05) (Figure 2A,B and Table S3). At the genus level, ten taxa in nasal were overabundant in CP children including *Granulicatella*, *Actinomyces*, *Atopobium*, *Gemella*, *Veillonella*, *Rothia*, *Prevotella*, *Haemophilus, Staphylococcus,* and *Streptococcus*, and five taxa in oral were overabundant in healthy children (Table S3). Interestingly, we found that bacterial biomarkers such as *Streptococcus*, *Gemella*, *Veillonella*, *Prevotella*, and *Haemophilus* in the nasal cavity of CP children were the dominant bacteria in the oral cavity (relative abundance > 1%). However, little or none of them occurred in the nasal cavity of healthy children in our data (Figure 1C and Table S3). Significantly, *Streptococcus* even became the third most abundant bacterial species in the nasal cavity of CP children. In general, all the results showed that some oral resident bacteria had an abnormally high abundance in the nasal cavity of CP children, which might significantly interfere with the nasal microecosystem.

### 2.2. Differences in Oral and Nasal Bacterial Communities between Healthy, CP, and Postoperative Adolescents

In order to assess the effect of CP repair on oronasal microecology, salivary and nasal samples of 5 CP adolescents, 10 postoperative adolescents (having undergone palatoplasty > 10 years previously), and 10 healthy adolescents at the same age (12–18) were collected for sequencing. There were no significant differences in mean age, gender composition, and DMFT between the three groups (Table S4).

#### 2.2.1. The Bacteria of Postoperative Adolescents Were Similar to CP Adolescents

**Figure 3:**
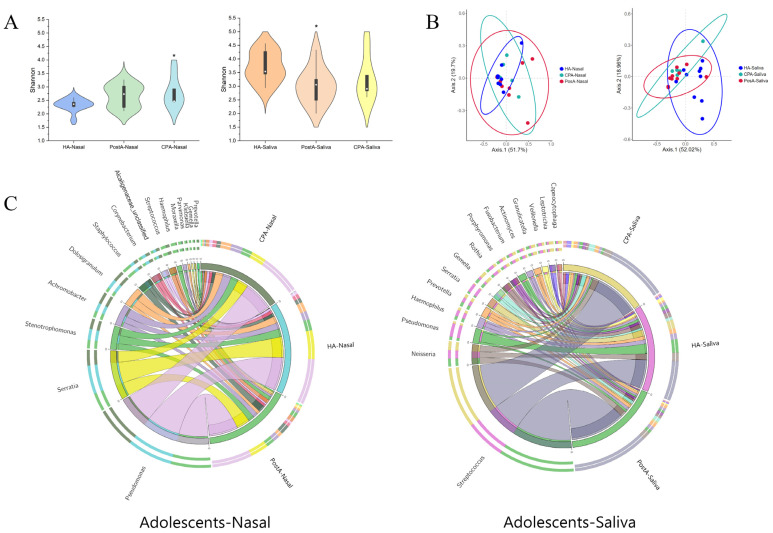
Characteristics of oral and nasal bacterial community in CP, postoperative and healthy adolescents. (**A**) Shannon index and (**B**) Principal coordinate analysis based on Bray–Curtis of salivary and nasal bacteria in the three groups. (**C**) The circlize demonstrates the community structure via the relative abundance of the top 15 species of salivary and nasal bacteria in the three groups. The *p* values were obtained by analysis of variance (* *p* < 0.05). HA: healthy adolescents. CPA: cleft palate adolescents. Post A: postoperative adolescents.

Figure 3A and Table S5 show the α diversity index of salivary and nasal bacteria in each group. Compared with healthy adolescents, the Shannon index of nasal bacteria in CP and postoperative adolescents was higher (2.82 vs. 2.33, *p* < 0.05; 2.66 vs. 2.33, *p* > 0.05). However, salivary bacteria showed an opposite trend (3.29 vs. 3.71, *p* > 0.05; 3.02 vs. 3.71, *p* < 0.05). Then, the similarity of the microbial community structures between the three groups was evaluated by PCoA (Figure 3B). For the nasal cavity, compared with the healthy group, the bacterial community structures of the CP and postoperative groups were more similar. For the oral cavity, salivary samples of these three groups exhibited a similar trend. As with children, cleft palate caused differences in the diversity of nasal cavity flora in healthy and unrepaired CP adolescents (Figure 3B and Table S5). Such difference was also shown between healthy and postoperative adolescents (Table S5).

#### 2.2.2. Taxonomic Composition of Nasal and Oral Bacterial Communities in CP, Postoperative, and Healthy Adolescents

**Figure 4:**
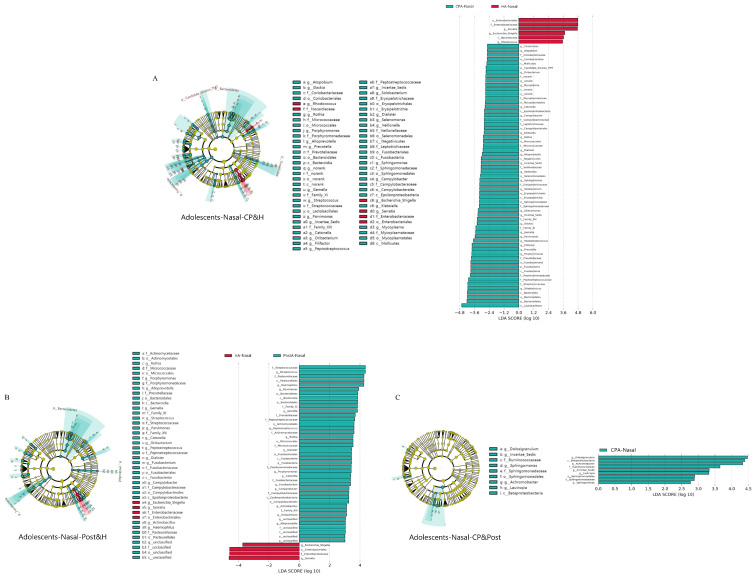
Distinct genera between the CP, postoperative, and healthy adolescents. (**A**–**C**) Linear discriminant analysis effect size (LEfSe, α = 0.05, log_10_ LDA score > 2.0) revealed 21 and 14 genera (green) with greater relative abundance in the CP and postoperative than the healthy group of nasal bacteria. There are only 4 genera with greater relative abundance in the CP group than postoperative group. H: healthy. CP: cleft palate. Post: postoperative.

Figure 3C shows the bacterial community structure at the genus level in each group. In detail, as for nasal bacteria, the top three genera in different groups were *Pseudomonas*, *Serratia*, and *Stenotrophomonas* in healthy adolescents; *Pseudomonas*, *Serratia*, and *Achromobacter* in postoperative adolescents; and *Pseudomonas*, *Serratia*, and *Staphylococcus* in CP adolescents. As for salivary bacteria, the top three genera in different groups were *Streptococcus*, *Pseudomonas*, and *Serratia* in healthy adolescents; *Streptococcus*, *Neisseria*, and *Prevotella* in postoperative adolescents; and *Streptococcus*, *Neisseria*, and *Gemella* in CP adolescents. LEfSe was also used to identify the special microbial biomarkers at the genus level in nasal and salivary bacteria in the three groups (Figure 4A–C, Tables S6 and S7). Importantly, in the nasal cavity, 21 genera in CP adolescents and 14 genera in postoperative adolescents showed higher abundance than in healthy adolescents (LDA score > 2.0, *p* < 0.05). Among them, there were 11 genera biomarkers shared by the two groups, including *Streptococcus*, *Gemella*, *Rothia*, *Alloprevotella*, *Campylobacter*, *Catonella*, *Porphyromonas*, *Dialister*, *Peptostreptococcus*, *Parvimonas*, and *Oribacterium*. Moreover, our data showed that *Atopobium*, *Rothia*, *Veillonella*, *Gemella*, and *Prevotella* belonging to common oral bacteria (relative abundance > 1%) were constant biomarkers of nasal microbe, with no difference in CP children, CP adolescents, or postoperative adolescents, implying that these biomarkers might have bacterial translocation between oral and nasal cavities. Moreover, it was independent of the growth and development or cleft palate repair.

### 2.3. Oral–Nasal Bacterial Translocation in CP Patients

**Figure 5:**
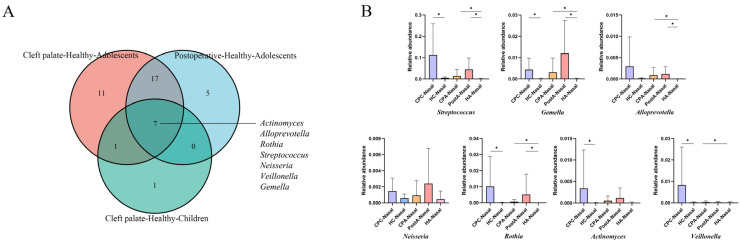
Characteristics of TS O-N bacs in children and adolescents. (**A**) Venn diagram revealed there were seven TS O-N bacs in children and adolescents. (**B**) Changes in relative abundance of seven TS O-N bacs in nasal of five populations (CP and healthy children; CP, healthy, and postoperative adolescents). The *p* values were obtained by LEfSe (* *p* < 0.05, α = 0.05, log_10_ LDA score > 2.0).

**Figure 6:**
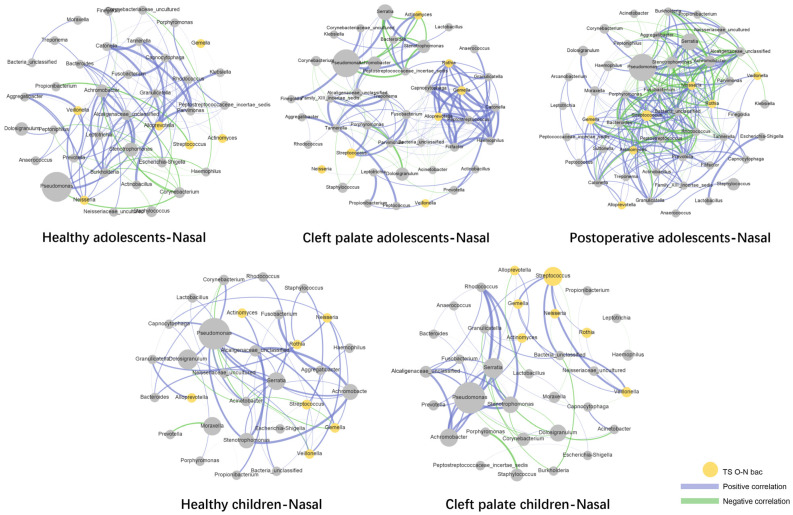
Correlation network analysis of nasal bacteria in five groups (CP and healthy children; CP, healthy, and postoperative adolescents). Correlation network analysis graphs contain nodes and edges. Nodes represent bacterial genera, and edges represent spearman correlation coefficient. Blue edges represent positive correlation and green represent negative correlation. Significant correlations (*p* < 0.05) with a coefficient of at least 0.6 are exhibited.

In both children and adolescents, our findings showed that some common oral bacteria occurred with abnormally high abundance in nasal microecology of CP subjects, indicating possible bacterial migration between the oral and nasal niches. Therefore, we attempted to confirm the specific taxon participating in and potentially inducing changes in the CP-related bacteria in the nasal cavity. Here, we defined several common oral bacteria as the oral–nasal translocation bacteria (O-N bac). They met the following conditions in our study: (1) genera colonized at a high rate in the oral cavity as reported [24,25], not found in the nasal cavity of the healthy group but found in the CP group; (2) the content of bacteria in the nasal cavity of the healthy group was <0.01%, while that in the saliva was >0.01%; (3) the bacteria were non-dominant (<1%) in the nasal cavity of the healthy group and dominant in the nasal cavity of the CP group (>1%). The definition of nasal–oral translocation bacteria (N-O bac) was as follows: (1) genera colonized at a high rate in the nasal cavity as reported, not found in the oral cavity of the healthy group but found in the CP group; (2) the content of bacteria in the oral cavity of the healthy group was <0.01%, while that in the nasal cavity was >0.01%; (3) the bacteria were nondominant (<1%) in the oral cavity of the healthy group and dominant in the oral cavity of the CP group (>1%). Our data showed that the nasal bacterial ecosystem was affected at a higher rate than that of the saliva by CP; therefore, we directed more attention to the nasal cavity and the O-N bacs. According to the criteria above, we screened a series of O-N bacs in CP children and CP adolescents, as shown in Tables S8 and S9. In CP children and adolescents, 9 and 36 genera were identified as O-N bacs, respectively. Interestingly, even if the cleft palate had been closed for >10 years, 29 O-N bacs were still retained in the postoperative adolescents, with 24 overlapping with the O-N bacs in the CP adolescents. Then we added the Table S10 of the oral dominant bacteria in each group with O-N bacs. In order to explore the temporal characteristics of these migrating bacteria, we took the intersection sets of O-N bacs selected from CP children, CP adolescents, and postoperative adolescents (Figure 5A). The bacteria at the intersection of the three were defined as TS O-N bacs. We speculated that these bacteria might migrate from the oral cavity to the nasal cavity through the cleft during childhood in CP patients and establish a bacterial community in the nasal cavity. Cleft palate repair also failed to reverse such bacterial community changes. Our results revealed that *Streptococcus*, *Gemella*, *Alloprevotella*, *Neisseria*, *Rothia*, *Actinomyces*, and *Veillonella* were TS O-N bacs (Figure 5A). Next, we primarily described changes in the relative abundance of TS O-N bacs in all the groups. All the TS O-N bacs showed higher relative abundance in CP-related nasal bacteria than healthy subjects, independent of the age or cleft palate repair (Figure 5B). Then, we carried out a network analysis (Figure 6), which demonstrated the interaction of bacteria in five nasal samples, with TS O-N bacs highlighted in yellow. The results showed that the correlation of nasal bacteria in adolescents was more complex than in children. Moreover, the microbial networks in the nasal cavity of CP and postoperative adolescents were more complicated compared with healthy individuals. Importantly, *Rothia*, *Gemella*, and *Alloprevotella*, some TS O-N bacs, developed a “solid” network structure in CP adolescents. Meanwhile, *Gemella*, *Streptococcus*, *Neisseria*, *Rothia*, and *Actinomyces* similarly served as central hubs and formed an intricate network relationship with other bacteria. 

## 3. Discussion

Landscape ecology considers that the potential spatial heterogeneity in geomorphic and environmental characteristics affect the structure and functional spatial pattern of the bacterial community. Bacteria from different ecological sites, such as the nose, mouth, and throat, communicate with each other or spread to the distal parts of the organism. For example, adenoids might become a reservoir of pathogens for middle ear infections through the eustachian tube [26]. Palate fractures result in morphological communication at the junction of ecological sites in the oronasal cavity. The structure and function of the flora are influenced by changes in the ecological landscape [27]. In our study, CP patients’ nasal and oral ecosystems were characterized by a degree of dysbiosis. There were some changes in microbial composition compared with healthy subjects. Consistent with previous findings [11], the relative abundance of common bacteria in children’s nasal cavities, such as *Dolosigranulum* and *Moraxella* [28], decreased, but that of some bacteria such as *Streptococcus* increased relatively.

This study showed that CP significantly affected the bacteria of the two ecological sites, and the effect on nasal bacterial flora was more significant, consistent with a previous work based on the polymerase-chain-reaction-denaturing gradient gel electrophoresis (PCR-DGGE) [11]. Zhou et al. investigated the temporal stability of 22 habitats by sequencing analysis. They found that the oral habitat had the most stable bacterial flora with the highest alpha diversity, which might be one reason why salivary bacteria of CP patients were not easily disturbed by the environment [29]. The community succession pattern in the nasal cavity is a substitute form, i.e., new species appear and replace the old species. For instance, *Staphylococcus* and *Corynebacterium* dominance in infancy was replaced by *Moraxella* or *Alloiocococus* spp. dominance with age. Various invaders possess different abilities to replace the dominant strains [30]. Such an easily replaced community might explain why CP influences the nasal cavity more significantly.

The composition and function of the bacteria play important roles in human health and disease [31]. The invasion of heterologous bacteria may disturb the balance and cause diseases [32]. In the digestive system, because of the impairment of intestinal barrier function in patients with liver cirrhosis or obesity, intestinal bacteria and their metabolites are transferred from the intestine to the blood or other organs, causing infection [33]. Recent studies have also revealed that the translocation of vaginal bacteria is associated with uterine health [32]. Pathogens might migrate from the oral cavity to all parts of the body. Systemic diseases, including atherosclerotic disease, rheumatoid arthritis, inflammatory bowel disease, and colorectal cancer, have been demonstrated to be associated with the ectopic colonization of oral pathogens [22,23]. Interestingly, we found that part of the nasal bacterial biomarkers in CP patients were common oral bacteria, indicating that certain oral bacteria might migrate through the enlarged cleft to the nasal cavity. According to our setting, we observed that 9, 36, and 29 genera called O-N bacs migrated from the oral cavity to the nasal cavity in CP children, CP adolescents, and postoperative adolescents, respectively. Especially, our results revealed that a large number of salivary bacteria such as *Streptococcus*, *Prevotella*, *Veillonella*, *Actinomyces*, etc., were colonized in the nasal cavity of CP patients. In previous studies, the increase in *Streptococcus* spp. and *Prevotella salivae* in nose/throat has been proved to be related to susceptibility to influenza virus infection [34]. *Veillonella dispar* was enriched in the salivary bacteria of chronic rhinosinusitis patients [35]. Furthermore, *Rothia*, *Actinomyces*, *Neisseria*, and *Veillonella* in the nasal cavity might be important in the causal pathway of otitis media [36]. In this study, increased colonization of the bacteria above in the nasal cavity might cause a dysbiosis of CP patients’ nasal microbiota and increase the possibility of patients suffering from infectious diseases. Although these genera are not considered pathogens of infectious diseases, they may be important in the causal pathway. Palate fracture enlarged the space of the oral and nasal connecting passage. We speculate that after sucking, swallowing, and other functions, bacteria were exchanged between the saliva and nasal secretions through the cleft palate in two sites, enhancing the possibility of bacterial exchange, ectopic colonization, and mutual influence in two habitats. However, further evidence is required to determine whether this migration of flora actually occurs and whether it will increase the incidence of respiratory diseases in patients with cleft palate.

Cleft palate repair can restore the normal structure and function of the oronasopharynx by closing the abnormal fissures [37]. At the same time, it can modify the ecological environment and affect the composition of bacteria. Prior works have identified the bacterial differences in nasal, sublingual, and oropharyngeal surfaces in CP patients before or after reconstructive surgery. Cleft palate closure significantly reduced *Klebsiella, Enterobacter species,* and *Staphylococcus aureus* counts [19,38]. We compared the oral and nasal bacteria of CP, postoperative, and healthy adolescents. However, the results demonstrated that compared to the healthy adolescents, the bacterial composition of saliva and the nasal cavity in the CP and postoperative adolescents was more similar. In addition, in the present study, changes in the bacterial community diversity and bacterial biomarkers identified by LEfSe showed that some changes in the flora caused by cleft palate in childhood were also reflected in adolescence, independent of cleft palate repair. Therefore, we speculated that the changes in microflora induced by CP in childhood had a far-reaching impact on the development of the host’s oral and nasal bacteria. To further explore the temporal characteristics of O-N bacs, we defined TS O-N bacs located at the intersection center of the O-N bacs in three categories, including *Streptococcus*, *Gemella*, *Alloprevotella*, *Neisseria*, *Rothia*, *Actinomyces*, and *Veillonella*. TS O-N bacs translocated from the oral cavity to the nasal cavity during childhood colonized the nasal cavity, possibly affecting the development of nasal microbiota and even becoming dominant in the CP adolescents’ nasal bacteria. Moreover, the effect of TS O-N bacs on nasal flora would not be greatly disturbed by cleft palate repair. Therefore, CP adolescents and postoperative adolescents had a relatively similar nasal ecosystem. More importantly, some of these TS O-N bacs, such as *Streptococcus*, *Rothia*, *Actinomyces*, *Neisseria*, and *Veillonella*, etc., were associated with respiratory tract infections and otitis media [34,35,36]. The study of O-N bacs and TS O-N bacs provides a new explanation for the dysbiosis of nasal microbiota in the CP patients; that is, such dysbiosis may come from the ectopic colonization of oral bacteria in the nasal cavity. Understanding the source of dysbiosis can help us to consider the etiology from multiple perspectives and provide combined treatment for CP patients suffering from respiratory infectious diseases. Nasal flora dysbiosis in CP patients may be one of the reasons for increased susceptibility to respiratory infectious diseases or otitis media. In fact, this hypothesis needs to be further explored in a case–control study in the CP patients with or without respiratory infectious diseases. The study of nasal and oral microbiota can help predict the risk of suffering from respiratory diseases in CP patients and prevent or treat diseases from the perspective of flora intervention.

However, there are still some limitations in this research. The genus “Serratia” was detected in most nasal samples and occupied a high relative abundance, which was different from the previous research results [39]. Serratia is usually detected in the environment [40], as well as in the oral cavity [41] and skin [42] of some specific people, but is mentioned in only two examples from the literature related to the nasal microbiome [43,44]. As for the source of Serratia, we proposed the following possibilities: (1) the high abundance of Serratia is one of the nasal microbiome characteristics in our subjects, because the composition of the nasal microbiome is affected by many factors, including age of subjects, region, climate, and individual differences [39,45,46,47]; (2) the sequencing platform we used was the Roche Genome Sequencer FLX + platform with 1% error rate [48]. We cannot rule out that this phenomenon may be caused by sequencing errors. Furthermore, due to the limitation of the sequencing platform (Roche Genome Sequencer FLX +), there was a 1% error rate in the sequencing results [48] and a low annotation rate at species level. Our analysis of the data was limited to the genus level. In the future, studies related to high-throughput sequencing of CP patients’ oral and nasal microbiome should be carried out on a platform with more optimized performance. In addition, this study is limited by the small number of subjects, so the results may not be applicable to other CP patients. It will be more scientific to investigate more samples in each group. 

## 4. Materials and Methods

### 4.1. Study Design and Procedures

This study was approved by the Medical Ethics Committee of West China Stomatology Hospital of Sichuan University (Protocol Number: WCHSIRB-ST-2013-069). Informed consent was obtained from the subjects and their legal guardians. The sample consisted of 45 individuals visiting West China Stomatology Hospital or West China Women’s and Children’s Hospital of Sichuan University, including 10 CP children, 10 healthy children, 5 CP adolescents, 10 postoperative adolescents, and 10 healthy adolescents. Children and adolescents with CP were in the corresponding age group (1–2 and 12–18 years old), diagnosed with nonsyndromic complete CP with or without a cleft lip, and had not undergone palatoplasty. The postoperative adolescents were diagnosed similarly and had undergone surgery > 10 years previously without complications. All subjects in postoperative group received palatoplasty at their 10m–1y. Exclusion criteria of children included: being non-Han; teeth have not yet erupted; presence of active oral or respiratory infectious diseases; presence of acute infectious diseases; presence of systemic diseases; having used antibiotic treatments in 3 months before the study. Exclusion criteria of adolescents included: being non-Han; being in mixed dentition; has received or is receiving removable or fixed orthodontic treatment; presence of oral or respiratory infectious diseases; presence of acute infectious diseases; presence of systemic diseases; having used antibiotic treatments in 3 months before the study; having complications such as palate fistula and dehiscence, etc., after cleft palate repair operation.

Demographic information (age, gender, and delivery mode) was collected from the subjects and their legal guardians by trained investigators. Diagnostic and surgical information was collected from the attending physician for these subjects. We performed a simple oral examination on the subjects. The incidence of teeth eruption of the included children was examined. The DMFT (decayed, missing, and filled teeth) index of adolescents was calculated by trained investigators. The relevant information of the subjects was displayed in Tables S1 and S3.

### 4.2. Sample Collection

Sampling methods conformed to the NIH human microbiology program (www.hmpdacc.org/doc/HMP_ClinicalProtocol.pdf, accessed on 30 March 2009). After fasting for 2 h before sampling, 1 mL of unstimulated saliva was collected from the oral cavity floor of the subjects with a disposable sterile pipette and transferred into the centrifuge tube. After gently rotating on the nasal cavity’s vestibular wall, the sterile nylon flocking swab was immersed in a centrifuge tube filled with 750 μL of PBS buffer and shaken for 10 s. Then, the swab was attached to the centrifuge tube wall several times. The swab was released into the buffer as much as possible. The collected specimens were frozen at −80 °C until use within 2 hours after transportation under an ice bath.

### 4.3. DNA Extraction and 16S rRNA Gene Amplicon Sequencing

Microbial DNA was extracted from 90 samples using the E.Z.N.A.® Soil DNA Kit (Omega Bio-Tek, Norcross, GA, USA). The V3–V6 region of the bacteria 16S ribosomal RNA gene was amplified by polymerase chain reaction (95 °C for 2 min, followed by 25 cycles at 95 °C for 30 s, 55 °C for 30 s, and 72 °C for 30 s, and a final extension at 72 °C for 5 min) using primers 341F:5’-CCTACGGGAGGCAGCAG-3’ and 1073R:5’-ACGAGCTGACGACARCCATG-3’. PCR reactions were performed in a 20 μL mixture containing 4 μL of ×5 FastPfu Buffer, 2 μL of 2.5 mm dNTPs, 0.8 μL of each primer (5 μM), 0.4 μL of FastPfu Polymerase, and 10 ng of template DNA. EmPCR products were prepared using the Roche emPCRAmp-Lib_L Kit (Roche, Indianapolis, IN, USA) designed by Roche and sequenced using GS FLX+_ Sequencing Method Manual_XL+ Kit (Roche, Indianapolis, IN, USA) on the Roche Genome Sequencer FLX + platform (Roche, Indianapolis, IN, USA). 

### 4.4. Bioinformatics Analysis and Statistical Analysis

The optimized sequence data were obtained by discarding sequences with lengths < 200 bp, a fuzzy base number > 0, and average sequence quality < 25.

Sequence bioinformatics analysis was performed using the Usearch analysis platform (version 7.1, http://drive5.com/uparse/, accessed on 8 July 2016). After extracting nonrepetitive sequences and removing single sequences, the sequences were clustered to operational taxonomical units (OTU) at a 97% similarity level. RDP Bayesian Classifier algorithm was applied to sequence homology alignment and species taxonomic identification of 97% similar OTU representative sequences. Qualified sequences were submitted to the Silva database (Release119, http://www.arb-silva.de, accessed on 8 July 2016) for taxonomic alignment. Alpha diversity ACE, Chao 1, and Shannon index were determined to evaluate the diversity and abundance of the community. Beta diversity (distance between samples based on differences in OTUs present in each sample) was measured using the Bray–Curtis distance. The relative abundance of the top 15 genera in each group was shown by circos diagrams. Linear discriminant analysis effect size (LEfSe) was used to determine the biomarker genera that best characterized each group. We considered taxa with a linear discriminant analysis score of > 2 and *p* < 0.05 significant. Correlation networks were developed based on microbial profiles at the genera level (Spearman’s correlation coefficient > 0.6, *p* < 0.05). Each genus was represented as a network node, and Spearman’s correlation coefficient was defined as the edge weight. Networks were visualized using Gephi (version 2021.0.9.2, Oracle, Austin, TX, USA). The average age, gender distribution, birth mode, and the number of erupted teeth in the child groups were compared by the Mann–Whitney U test and Fisher’s exact test, and the diversity indexes were tested by t-test. The average age, gender distribution, and DMFT in adolescent groups were compared by the independent-sample Kruskal–Wallis test and Fisher’s exact test, and the diversity indexes were tested by one-way ANOVA. We set the *p*-value threshold at 0.05. Statistical analyses were carried out by SPSS (version 19.0, IBM, Armonk, NY, USA). Graphpad Prism8 (GraphPad Software, San Diego, CA, USA) and Oringin2021b (OriginLab, Northampton, MA, USA) were used for some drawings.

## Data Availability

The 16S rRNA gene sequence datasets are available in the NCBI repository with the BioProject ID: PRJNA846104.

## Supplementary Materials

The following supporting information can be downloaded at: https://www.mdpi.com/article/10.3390/pathogens11070771/s1, Table S1: Basic information of included children, Table S2: Comparison of α-diversity index in oral and nasal samples of two groups in children, Table S3: Basic information of included adolescents, Table S4: Comparison of α-diversity index in oral and nasal samples of three adolescence groups, Table S5: Microbial biomarkers of oronasal bacteria of children, Table S6: Microbial biomarkers in the nasal cavity of adolescents, Table S7: Microbial biomarkers in the saliva of adolescents, Table S8: Translocation bacteria between oral and nasal cavity CP children, Table S9: Translocation bacteria between oral and nasal cavity in CP adolescents, Table S10: Dominant oral bacteria (>1%) in each group.
