# Supplementary material for: Characterization of Bacterial Differences Induced by Cleft-Palate-Related Spatial Heterogeneity
Source: Pathogens. 2022 Jul 5;11(7):771. doi: 10.3390/pathogens11070771 (PMC9323727; doi:10.3390/pathogens11070771)
Supplement: Supplementary file 1 [file pathogens-11-00771-s001.zip › pathogens-1743739-supplementary.pdf]

Supplementary materials

Table S1. Basic information of included children

Number	Diagnosis	Gender	Delivery	Age (month)		Number of erupted teeth	
A1	RCCL, RCCP, RCCA	M	N	19	13.2±2.3	12	5.0±2.9
A2	BCCP	M	N	12		4	
A3	BCCL after surgery, BCCP	M	C	12		3	
A4	BCCL after surgery, BCLA, BCCP	F	C	15		7	
A5	RCL after surgery, RCLA, BCCP	M	N	14		7	
A6	BCCP	F	C	12		3	
A7	BCCP	F	C	12		3	
A8	BCL after surgery, LCCA, LCCP	M	N	12		4	
A9	LCCA, LCL, LCCP	F	N	12		4	
A10	RCCA, RCL after surgery, RCCP	M	N	12		3	
B1	Control	M	C	12	12.3±0.9	4	4.5±2.1
B2	Control	F	N	12		4	
B3	Control	M	N	12		5	
B4	Control	F	C	12		3	
B5	Control	M	N	12		5	
B6	Control	M	N	12		4	
B7	Control	F	C	15		8	
B8	Control	M	C	12		8	
B9	Control	M	N	12		2	
B10	Control	F	N	12		2	
	P value	1.000[2]	1.000[2]	0.279[1]		0.969[1]	

A1-A10: cleft palate children; B1-B10: healthy children

Diagnostic name notes: CCP, complete cleft palate; CCA, complete cleft alveolar; CCL, complete cleft lip; B, both sides; R, right side; L, left side.

Gender notes: M, male; F, female.

Delivery mode: N, natural labor; C, caesarean birth.

[1] Test by Mann-Whitney U; [2] Test by Fisher's exact

Table S2. Comparison of α-diversity index in oral and nasal samples of two groups in children

Diversity index (M ± SD)	CP group		Healthy group	
	Saliva	Nasal	Saliva	Nasal
Ace	195.70±42.88 [b]	155.6±24.70 [b]	203.10±39.00 [c]	129.90±45.33 [c]
Chao1	196.80±43.43 [b]	140.20±17.42 [ab]	198.10±30.49 [c]	114.60±29.20 [ac]
Shannon	2.53±0.49	2.68±0.21 [a]	2.47±0.46	2.18±0.31 [a]

SD: Standard deviation, CP: Cleft palate

[a]There was significant difference in nasal microbiota between the two groups（p <0.05）

[b]There was significant difference between salivary and nasal microbiota in CP group（p <0.05）

[c]There was significant difference between salivary and nasal microbiota in healthy group（p <0.05）

Table S3. Microbial biomarkers of oronasal bacteria of children

Microbial biomarkers of children	
Nasal	Saliva
Cleft palate > Healthy	Healthy > Cleft palate
Granulicatella	*Haemophilus*
Actinomyces[*†]	*Pseudomonas*
Atopobium	*Aggregatibacter*
Gemella[*†]	*Serratia*
Veillonella[*†]	*Achromobacter*
Rothia[*†]	
Prevotella[*]	
Haemophilus	
Staphylococcus	
Streptococcus[*†]	

[*]O-N bacs in the nasal microbial biomarkers of CP children

[†]TS O-N bacs in the nasal microbial biomarkers of CP children

Table S4. Basic information of included adolescents

Number	Diagnosis	Palatoplasty (yes/no)	Gender	Age (year)		DMFT	
C1	BCCP	no	M	15		0	
C2	LCCL after surgery， LCCA， LCCP	no	M	18		0	
C3	LCCL after surgery， LCCA， LCCP	no	M	18	16.4±2.3	1	0.2±0.4
C4	LCCP	no	M	18		0	
C5	RCCL after surgery， RCCA， RCCP	no	F	13		0	
D1	BCCL after surgery，BCCP after surgery， BCCA	yes	M	13		3	
D2	LCCL after surgery，LCCP after surgery， LCCA	yes	F	18		0	
D3	LCCL after surgery，LCCP after surgery， LCCA	yes	M	18		0	
D4	LCCP after surgery， LCCA	yes	M	15		0	
D5	BCCL after surgery，BCCP after surgery BCCA	yes	M	16		2	
D6	BCCL after surgery，BCCP after surgery BCCA	yes	M	18	16.2±1.8	0	0.7±1.2
D7	BCCP after surgery	yes	M	16		0	
D8	LCCL after surgery，LCCP after surgery LCCA	yes	F	15		0	
D9	LCCL after surgery，LCCP after surgery LCCA	yes	M	15		0	
D10	RCCP after surgery， RCCA	yes	M	18		2	
E1	Control	-	M	16		0	
E2	Control	-	F	18		0	
E3	Control	-	F	17		0	
E4	Control	-	F	18		0	
E5	Control	-	M	14	16.5±1.8	2	0.3±0.7
E6	Control	-	M	14		0	
E7	Control	-	M	14		1	
E8	Control	-	M	18		0	
E9	Control	-	M	18		0	
E10	Control	-	M	18		0	
	P		1.000[2]	0.873[1]		0.618[1]	

C1-C5: cleft palate adolescents; D1-D10: postoperative adolescents; E1-E10: healthy adolescents

Diagnostic name notes: CCP, complete cleft palate; CCA, complete cleft alveolar; CCL, complete cleft lip; B, both sides; R, right side; L, left side.

Gender notes: M, male; F, female.

[1] Test by independent-sample Kruskal-Wallis test；

[2] Test by Fisher's exact

Table S5. Comparison of α-diversity index in oral and nasal samples of three adolescence groups

		Nasal		Saliva	
		**M**	**SD**	**M**	**SD**
**Ace**	CP group	219.4[a]	83.54	346.8	46.27
	Postoperative group	180.6[a]	78.61	332	44.81
	Healthy group	113.6	49.16	337	96.01
**Chao1**	CP group	182.2[a]	72.95	342	37.98
	Postoperative group	161.9[a]	67.52	307.7	50.15
	Healthy group	106.2	33.83	331.5	91.32
**Shannon**	CP group	2.82[a]	0.58	3.29	0.85
	Postoperative group	2.66	0.42	3.02[b]	0.67
	Healthy group	2.33	0.24	3.71	0.56

[a]There was significant difference in nasal microbiota between the tag and healthy group （p<0.05）

[b]There was significant difference in salivary microbiota between the tag and healthy group （p<0.05）

Table S6. Microbial biomarkers in the nasal cavity of adolescents

Microbial biomarkers in the nasal cavity of adolescents				
Cleft palate-Healthy		Postoperative-Healthy		Cleft palate-Postoperative
Cleft palate > Healthy	Healthy > Cleft palate	Postoperative > Healthy	Healthy > Postoperative	Cleft palate > Postoperative
Atopobium[※]	*Serratia*	*Streptococcus*[#†]	*Escherichia_Shigella*	*Dolosigranulum*
Oribacterium[※]	*Escherichia_Shigella*	*Haemophilus*[#]	*Serratia*	*Achromobacter*
Mycoplasma[※]	*Rhodococcus*	*Parvimonas*[#]		*Lautropia*
Catonella[※]		*Gemella*[#†]		*Sphingomonas*
Campylobacter[※]		*Peptostreptococcus*[#]		
Klebsiella		*Rothia*[#†]		
Rothia[※†]		*Dialister*[#]		
Dialister[※]		*Porphyromonas*		
Alloprevotella[※†]		*Catonella*[#]		
Veillonella[※†]		*Fusobacterium*		
Sphingomonas		*Campylobacter*[#]		
Solobacterium		*Actinobacillus*[#]		
Selenomonas[※]		*Oribacterium*[#]		
Slackia		*Alloprevotella*[#†]		
Gemella[※†]				
Parvimonas[※]				
Peptostreptococcus[※]				
Filifactor[※]				
Prevotella[※]				
Porphyromonas[※]				
Streptococcus[※†]				

[※]O-N bacs in the nasal microbial biomarkers of CP adolescents

[#]O-N bacs in the nasal microbial biomarkers of postoperative adolescents

[†]TS O-N bacs in the nasal microbial biomarkers of CP adolescents and postoperative adolescents

Table S7. Microbial biomarkers in the saliva of adolescents

Microbial biomarkers in the saliva of adolescents			
Cleft palate-Healthy	Postoperative - Healthy		Cleft palate- Postoperative
Healthy > Cleft palate	Postoperative > Healthy	Healthy > Postoperative	Cleft palate > Postoperative
Pseudomonas	*Streptococcus*	*Achromobacter*	*Campylobacter*
Serratia	*Granulicatella*	*Stenotrophomonas*	*Leptotrichia*
Stenotrophomonas		*Veillonella*	
Achromobacter		*Solobacterium*	
Kingella		*Serratia*	
Rhodococcus		*Burkholderia*	
		Pseudomonas	

Table S8. Translocation bacteria between oral and nasal cavity CP children

Direction	Genera	Proportion（%）			
		CP-nasal	H-nasal	CP-saliva	H-saliva
Nasal—Oral	*Cyanobacteria_norank*	0.023%	0.003%	0.012%	0.002%
	Dolosigranulum	8.865%	15.488%	0.015%	0.000%
	Staphylococcus	4.132%	0.048%	0.012%	0.002%
	Acinetobacter	0.017%	0.037%	0.008%	0.000%
Oral—Nasal	*Actinomyces*	0.340%	0.005%	1.347%	0.503%
	Prevotella	0.987%	0.008%	2.993%	1.238%
	Rothia	1.018%	0.013%	1.262%	0.983%
	Streptococcus	11.300%	0.502%	57.372%	54.840%
	Neisseria	0.147%	0.058%	9.580%	7.768%
	Veillonella	0.833%	0.027%	3.675%	3.943%
	Gemella	0.432%	0.017%	3.883%	2.332%
	Alloprevotella	0.293%	0.015%	2.962%	4.272%
	Lautropia	0.005%	0.000%	0.338%	0.098%

Nasal-Oral: Common nasal bacteria translocated to oral microbiota

Oral-Nasal: Common oral bacteria translocated to nasal microbiota

CP: Cleft palate group

H: Health group

Table S9. Translocation bacteria between oral and nasal cavity in CP adolescents

Direction	Genera	Proportion（%）					
		CP-nasal	Post-nasal	H-nasal	CP-saliva	Post-saliva	H-saliva
Nasal—Oral	*Staphylococcus*	3.420%	7.573%	3.868%	0.040%	0.022%	0.002%
	Corynebacterium	3.397%	2.792%	3.868%	1.147%	0.560%	0.913%
Oral—Nasal	*Actinobacillus*	0.037%	0.095%	0.003%	1.410%	0.470%	0.352%
	Actinomyces	0.103%	0.113%	0.008%	1.230%	2.103%	2.637%
	Alloprevotella	0.177%	0.118%	0.002%	0.407%	0.548%	0.340%
	Atopobium	0.027%	0.020%	0.000%	0.033%	0.067%	0.060%
	Campylobacter	0.097%	0.032%	0.000%	0.183%	0.130%	0.157%
	Catonella	0.083%	0.050%	0.002%	0.177%	0.298%	0.233%
	Dialister	0.017%	0.017%	0.000%	0.060%	0.045%	0.038%
	Family_XIII_incertae_sedis	0.430%	0.028%	0.000%	0.143%	0.128%	0.222%
	Filifactor	1.347%	0.040%	0.000%	0.173%	0.102%	0.202%
	Lachnoanaerobaculum	0.030%	0.035%	0.003%	0.927%	0.327%	0.502%
	Leptotrichia	0.040%	0.067%	0.003%	3.433%	0.743%	1.430%
	Mogibacterium	0.013%	0.012%	0.000%	0.017%	0.010%	0.025%
	Mycoplasma	0.013%	0.020%	0.000%	0.017%	0.003%	0.012%
	Oribacterium	0.063%	0.027%	0.000%	0.277%	0.487%	0.470%
	Parvimonas	0.677%	1.662%	0.002%	0.067%	0.070%	0.088%
	Peptostreptococcaceae_incertae_sedis	0.110%	0.025%	0.003%	0.130%	0.055%	0.228%
	Peptostreptococcus	0.953%	0.800%	0.000%	0.167%	0.210%	0.512%
	Rothia	0.137%	0.510%	0.000%	2.093%	2.245%	4.073%
	Tannerella	0.140%	0.017%	0.002%	0.793%	0.140%	0.183%
	Treponema	0.133%	0.023%	0.002%	0.287%	0.137%	0.353%
	Streptococcus	2.850%	4.412%	0.100%	45.413%	56.365%	29.637%
	Gemella	0.627%	1.207%	0.012%	4.663%	3.263%	2.208%
	Neisseria	0.187%	0.238%	0.045%	6.343%	5.913%	6.130%
	Veillonella	0.070%	0.025%	0.020%	1.740%	1.095%	2.803%
	Bergeyella	0.020%	0.008%	0.002%	0.750%	1.475%	1.028%
	Candidate_division_TM7_norank	0.027%	0.007%	0.000%	0.767%	0.133%	0.632%
	Eikenella	0.023%	0.008%	0.008%	0.233%	0.108%	0.120%
	Family_XIII_uncultured	0.073%	0.005%	0.000%	0.017%	0.023%	0.163%
	Johnsonella	0.123%	0.010%	0.002%	0.557%	0.105%	0.160%
	Peptococcus	0.247%	0.003%	0.000%	0.050%	0.017%	0.045%
	Pseudoramibacter	0.033%	0.000%	0.000%	0.007%	0.005%	0.008%
	Selenomonas	0.020%	0.005%	0.002%	0.763%	0.212%	0.670%
	Synergistaceae_uncultured	0.023%	0.003%	0.000%	0.030%	0.025%	0.018%
	Fusobacterium	1.503%	0.400%	0.017%	2.687%	1.698%	2.800%
	Porphyromonas	1.380%	0.405%	0.013%	2.500%	2.233%	2.577%
	Prevotella	1.320%	0.747%	0.052%	2.757%	3.382%	3.637%
	Abiotrophia	0.003%	0.017%	0.002%	0.720%	0.355%	0.347%

Actinobaculum	0.003%	0.012%	0.000%	0.063%	0.143%	0.027%
Aggregatibacter	0.003%	0.043%	0.007%	0.600%	0.398%	0.552%
Neisseriaceae_norank	0.000%	0.023%	0.003%	0.020%	0.205%	0.385%
Haemophilus	0.657%	4.183%	0.072%	2.077%	2.960%	4.417%

Nasal-Oral: Common nasal bacteria translocated to oral microbiota

Oral-Nasal: Common oral bacteria translocated to nasal microbiota

CP: Cleft palate group

H: Health group

Post: Postoperative group

In the oral-nasal part, black font: Exchange genera of CP and postoperative adolescents

Red font: Exchange genera of CP adolescents only

Green font: Exchange genera of postoperative adolescents only

Table S10. Dominant oral bacteria (> 1%) in each group

Children		Adolescents		
CP-saliva	H-saliva	Post-saliva	CP-saliva	H-saliva
Streptococcus(57.37%)*†	*Streptococcus*(54.84%)	*Streptococcus*(56.37%)※†	*Streptococcus*(45.41%)#†	*Streptococcus*(29.6%)
Neisseria(9.58%)*†	*Neisseria*(7.77%)	*Neisseria*(5.91%)※†	*Neisseria*(6.34%)#†	*Pseudomonas*(10.00%)
Granulicatella(4.12%)	*Granulicatella*(5.57%)	*Prevotella*(3.38%)	*Gemella*(4.66%)#†	*Serratia*(6.64%)
Gemella(3.88%)*†	*Bergeyella*(4.66%)	*Gemella*(3.26%)※†	*Leptotrichia*(3.43%)#	*Neisseria*(6.13%)
Veillonella(3.68%)*†	*Alloprevotella*(4.27%)	*Haemophilus*(2.96%)※	*Granulicatella*(3%)	*Haemophilus*(4.42%)
Prevotella(2.99%)*	*Veillonella*(3.94%)	*Granulicatella*(2.58%)	*Prevotella*(2.76%)#	*Rothia*(4.07%)
Alloprevotella(2.96%)*†	*Haemophilus*(3.17%)	*Pseudomonas*(2.37%)	*Fusobacterium*(2.69%)#	*Prevotella*(3.64%)
Bergeyella(2.57%)	*Porphyromonas*(2.34%)	*Rothia*(2.25%)※†	*Capnocytophaga*(2.63%)	*Veillonella*(2.80%)
Porphyromonas(1.75%)	*Gemella*(2.33%)	*Porphyromonas*(2.23%)	*Porphyromonas*(2.5%)#	*Fusobacterium*(2.80%)
Actinomyces(1.35%)*†	*Pseudomonas*(1.95%)	*Actinomyces*(2.10%)※†	*Rothia*(2.09%)#†	*Actinomyces*(2.64%)
Rothia(1.26%)*†	*Prevotella*(1.24%)	*Fusobacterium*(1.70%)	*Haemophilus*(2.08%)	*Porphyromonas*(2.58%)
Haemophilus(1.01%)		*Bergeyella*(1.48%)	*Veillonella*(1.74%)#†	*Stenotrophomonas*(2.40%)
		Serratia(1.38%)	*Pseudomonas*(1.66%)	*Gemella*(2.21%)
		Veillonella(1.10%)※†	*Actinobacillus*(1.41%)#	*Achromobacter*(1.82%)
			Actinomyces(1.23%)#†	*Lautropia*(1.59%)
			Corynebacterium(1.15%)	*Leptotrichia*(1.43%)
				Capnocytophaga(1.41%)
				Granulicatella(1.25%)
				Bergeyella(1.03%)

*O-N bacs in the oral dominant bacteria of CP children

※O-N bacs in the oral dominant bacteria of postoperative adolescents

#O-N bacs in the oral dominant bacteria of CP adolescents

†TS O-N bacs in the oral dominant bacteria

CP: Cleft palate group

H: Health group

Post: Postoperative group
